# The Nobel prize in physiology and medicine – 2022

**DOI:** 10.1007/s11224-023-02124-0

**Published:** 2023-02-07

**Authors:** Krisztina Hagymási

**Affiliations:** Department of Surgery, Transplantation and Gastroenterology, Semmelweis University, Budapest, Hungary

**Keywords:** Nobel Prize, Genome, Neanderthal, Denisovan, Paleogenomics

## Abstract

The Nobel Assembly at Karolinska Institutet awarded the 2022 Nobel Prize in Physiology or Medicine to a Swedish geneticist, Svante Pääbo, for his discoveries concerning the genomes of extinct hominins and human evolution, for the sequencing of the genome of the Neanderthal, the discovery of a previously unknown hominin, Denisova, and the establishment of a new scientific discipline, paleogenomics.

Svante Pääbo was born in Stockholm, Sweden, in 1955. He specializes in evolutionary genetics and earned his PhD in 1986 at Uppsala University for research investigating how the E19 protein of adenoviruses modulates the cell-surface expression of class I antigens, elucidating the effects of E19 on the immune system. He was a postdoctoral fellow at University of Zürich, Switzerland, later at University of California, Berkeley, USA, joining Allan Wilson’s laboratory and worked on the genome of extinct mammals. In 1990, he returned to Europe to become professor of general biology at the University of Munich, Germany. In 1999, he founded the Max Planck Institute for Evolutionary Anthropology in Leipzig, Germany, where he is still active. He is also an adjunct professor at Okinawa Institute of Science and Technology, Japan [1, 2] (Fig. 1).

**Fig. 1:**
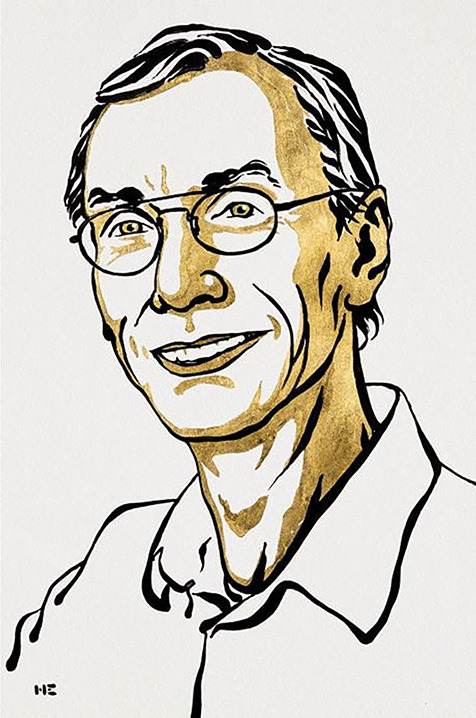
Svante Pääbo [1]

With time, DNA becomes chemically modified and degrades into short fragments. After thousands of years, the trace amounts of the left DNA are massively contaminated with DNA from bacteria and contemporary humans. His demonstration that DNA survived in the cell nuclei of some Egyptian mummies was published in 1984. Thereafter, he has developed techniques and approaches that allow DNA sequences from archaeological and paleontological remains to be determined. This has allowed ancient DNA from extinct organisms, humans, animals, and pathogens to be studied [1].

He sequenced the genome of the Neanderthal, an extinct relative of present-day humans. He also made the discovery of a previously unknown hominin, Denisova. It was the first time that a new hominin had been identified by genetic analysis alone. Pääbo also found that gene transfer, affecting how our immune system work, had occurred from these now lost hominins to *Homo sapiens* following the migration out of Africa around 70,000 years ago [1, 3].

Pääbo’s research gave rise to an entirely new scientific discipline; *paleogenomics*, a field of science based on the reconstruction and analysis of genomic information in extinct species.

Pääbo received several honors and awards for his discoveries in addition to the 2022 Nobel Prize, the Genetics Prize of the Gruber Foundation (2013), the Breakthrough Prize in Life Sciences (2016), and the Linnean Society of London’s Darwin-Wallace Medal (2019). He also became a member of the Royal Swedish Academy of Sciences (2000) and a foreign member of the National Academy of Sciences (2004) and the American Academy of Arts and Sciences (2011) [4].

## Studying the DNA of Neanderthals

DNA is localized in two different compartments in the cell. Nuclear DNA harbors most of the genetic information, while the much smaller mitochondrial genome is present in thousands of copies. Studying the DNA of Neanderthals is challenging, because with time DNA becomes chemically modified and degrades into short fragments, contaminated with DNA from bacteria and contemporary humans. Svante Pääbo developed methods for the retrieval of DNA sequences from archaeological and paleontological remains.

In 1990, Pääbo decided to analyze DNA from Neanderthal mitochondria—organelles in cells that contain their own DNA. mtDNA is conserved across eukaryotic organism given the critical role of mitochondria in cellular respiration. However, due to less efficient DNA repair (compared to nuclear DNA), it has a relatively high mutation rate (but slow compared to other DNA regions such as microsatellites) which makes it useful for studying the evolutionary relationships—phylogeny—of organisms. Pääbo managed to sequence a region of mitochondrial DNA from a 40,000-year-old piece of bone. The results of which discovered that humans (*Homo sapiens*) and Neanderthals (*H. neanderthalensis*) are distinct species that diverged from one another by about 500,000 years ago. Neanderthals have a significantly greater proportion of nonsynonymous to synonymous mutations in their sequences of the protein-coding mitochondrial genes. Moreover, the Neanderthal mitochondrial lineage was 20% shorter than a human one. As the sequence in question diverged from the common genetic line between humans and Neanderthals, the Neanderthals did not contribute to the mitochondrial genome of today’s humans [1, 5].

## Sequencing the Neanderthal genome

As analyses of the small mitochondrial genome gave only limited information, it represents a mere 0.0005% of a complete hominid genome and, moreover, is inherited strictly through the female lineage, Pääbo took on the challenge of sequencing the Neanderthal nuclear genome. At this time, he established the Max Planck Institute in Leipzig, Germany. With his research team achieved new technical developments with sequencing of DNA. They could publish the first Neanderthal genome sequence in 2010. Comparative analyses demonstrated that the most recent common ancestor of Neanderthals and *Homo sapiens* lived around 800,000 years ago. The presented draft sequence of the Neandertal genome composed of more than 4 billion nucleotides from three individuals. Comparisons of the Neandertal genome to the genomes of five present-day humans from different parts of the world identify a number of genomic regions that may have been affected by positive selection in ancestral modern humans. These genes are involved in metabolism and in cognitive and skeletal development [6].

Comparative analyses showed that DNA sequences from Neanderthals were more similar to sequences from contemporary humans originating from Europe or Asia than to contemporary humans originating from Africa. They suggested that gene flow took place when humans and Neanderthals co-occurred in the Levant, during the colonization process of Eurasia by modern humans. In modern-day humans with European or Asian descent, approximately 1–4% of the genome originates from the Neanderthals. The best estimates for genetic admixture from archaic hominids into the modern human gene pool were about 14% for Europeans and 1.5% for East Asians [1, 3].

## The discovery of Denisovan

In 2008, a 40,000-year-old fragment from a juvenile female finger bone was discovered in the Denisova cave in the southern part of Siberia. It is named after Denis (Dyonisiy), a Russian *hermit* who lived there in the eighteenth century. The well-preserved DNA, obtained from the bone, and sequenced by Pääbo’s team, indicates close affinities with Neanderthals, but the DNA sequence was unique when compared to all known sequences from Neanderthals and present-day humans. Pääbo had discovered a previously unknown hominin, which was given the name Denisovan. A tooth found in Denisova Cave as well carries a mitochondrial genome highly similar to that of the finger bone. This tooth shares no derived morphological features with Neanderthals or modern humans, further indicating that Denisovans are evolutionary distinct from Neanderthals and modern humans [7].

DNA evidence suggests they had dark skin, eyes, and hair, and had a Neanderthal-like build and facial features. Comparisons with sequences from contemporary humans from different parts of the world showed that gene flow had also occurred between Denisovans and *Homo sapiens*. Denisovans apparently interbred with modern humans, with the highest percentages (roughly 5%) occurring in Melanesians, Aboriginal Australians, and Filipino Negritos. This distribution suggests that there were Denisovan populations across Eurasia, the Philippines, and New Guinea and/or Australia [1, 7].

**Fig. 2:**
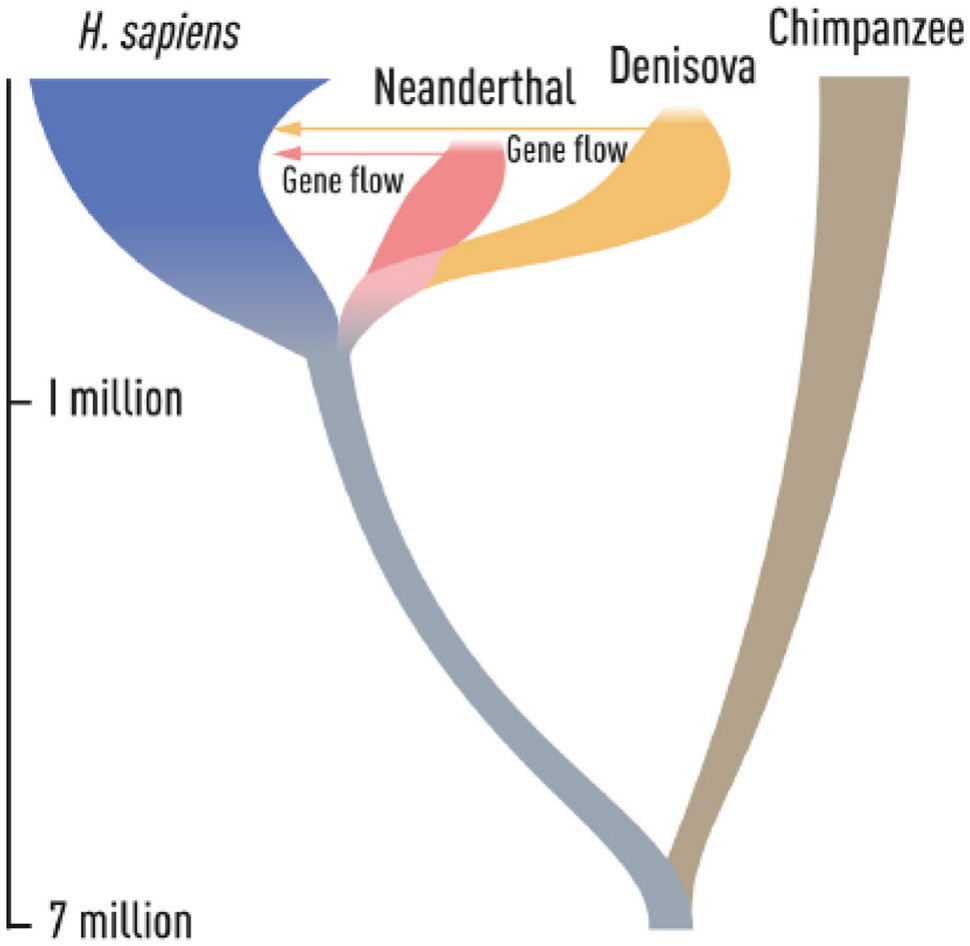
Phylogenetic tree demonstrating the evolution, relationship, and gene flow between Neanderthals and Denisovans [1]

At the time when *Homo sapiens* migrated out of Africa, at least two extinct hominin populations inhabited Eurasia. Neanderthals lived in western Eurasia, whereas Denisovans populated the eastern parts of the continent. During the expansion of *Homo sapiens* outside Africa, they encountered and interbred with Neanderthals, and with Denisovans as well (Fig. 2).

## Paleogenomics and its relevance

Through his seminal research, Svante Pääbo established an entirely new scientific discipline, *paleogenomics*. His research group has completed analyses of several additional genome sequences from extinct hominins.

**Fig. 3:**
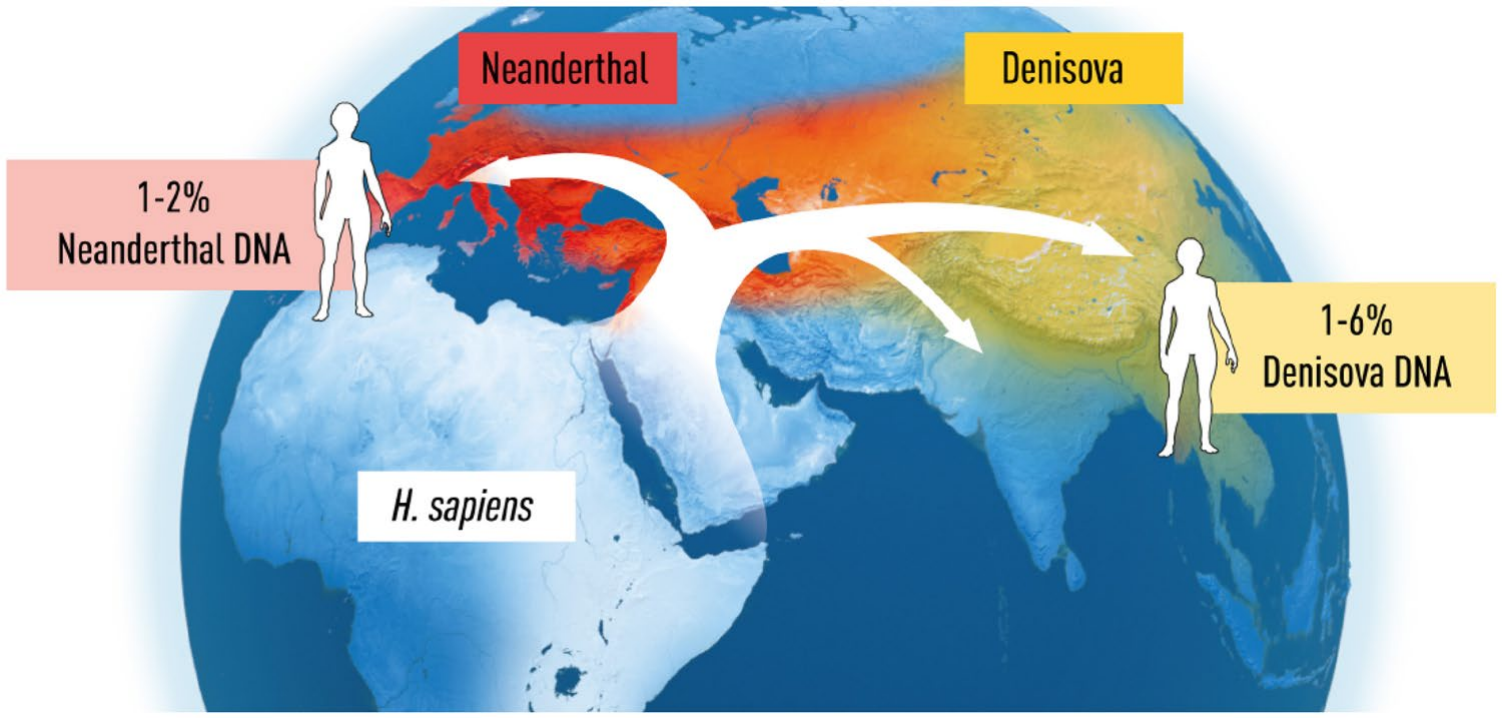
During the expansion of *Homo sapiens* outside Africa, they encountered and interbred with Neanderthals, and with Denisovans as well, resulting in genetic introgression [1]

Neanderthals and Denisovans died out for reasons that are not clear, but we know that archaic gene sequences from our extinct relatives influence the physiology of present-day humans (Fig. 3).

Introgression of archaic segments were beneficial for modern human adaptation a process known as “adaptive introgression.” One such example is the Denisovan version of the gene *EPAS1*, encoding major components of the hypoxia-inducible factor transcriptional system, which has a central role in oxygen sensing and coordinating an organism’s response to hypoxia, which confers an advantage for survival at high altitude and is common among present-day Tibetans [8].

Other examples are Neanderthal genes that affect our immune response to different types of infections. *OAS1* (transferred to the human genome from Neanderthals) is an interferon-induced gene, which encodes a protein that synthesizes 2′,5′-oligoadenylates (2-5As), playing a key role in innate cellular antiviral response, and has been implicated in other cellular processes like cell growth and apoptosis. Polymorphisms in this gene have been associated with susceptibility to viral infection, including SARS-CoV-2, and diabetes mellitus, type 1 [9].

*FOXP2* gene in Neandertals was identical to that of present-day humans. The protein is a transcription factors, regulates gene expression by binding to DNA. It is expressed in the brain, heart, lungs, and the digestive system. *FOXP2* was the first gene to be clearly linked to speech and language development in humans (“The language gene”). In humans, mutations in *FOXP2* cause the severe speech and language disorder, developmental verbal dyspraxia, characterized by difficulties in coordinating sequences of articulatory movements underlying proficient speech. There was no evidence of recent positive evolutionary selection of *FOXP2* in humans [10].

## Pääbo and COVID-19 infection

Several studies have shown that archaic introgression has affected human immune functions, host defense, making possible genetic adaptation of modern humans to the new pathogens.

Since first appearing in late 2019, the novel virus SARS-CoV-2 has had a range of impacts on those it infects. Some people become severely ill with COVID-19, with the requirement of hospitalization, whereas others have mild symptoms or are even asymptomatic. Professor Pääbo, in collaboration with Professor Hugo Zeberg, a researcher at the Max Planck Institute for Evolutionary Anthropology and Karolinska Institute, identified the genetic variants at chromosomal region 3, which spans 50 kb and contains six genes, features that are associated with European Neanderthal heritage, which have up to three times the risk of requiring mechanical ventilation.

They also showed that a haplotype at a region on chromosome 12 associated with requiring intensive care when infected with the virus is inherited from Neandertals. This region (OAS1, OAS2, OAS3) encodes proteins, induced by interferon, oligoadenylate synthetases, that are important during infections with RNA viruses, inducing antiviral mechanisms, that is why this Neandertal haplotype acts as a protective agent against severe disease [11, 12].

## Data Availability

Data sharing not applicable to this article as no datasets were generated or analyzed during the current study.
